# *Candida albicans* Cdc15 is essential for mitotic exit and cytokinesis

**DOI:** 10.1038/s41598-018-27157-y

**Published:** 2018-06-11

**Authors:** Steven Bates

**Affiliations:** Biosciences, College of Life and Environmental Sciences, University of Exeter, Exeter, EX4 4QD UK

## Abstract

*Candida albicans* displays a variety of morphological forms, and the ability to switch forms must be linked with cell cycle control. In budding yeast the Mitotic Exit Network (MEN) acts to drive mitotic exit and signal for cytokinesis and cell separation. However, previous reports on the MEN in *C*. *albicans* have raised questions on its role in this organism, with the components analysed to date demonstrating differing levels of importance in the processes of mitotic exit, cytokinesis and cell separation. This work focuses on the role of the Cdc15 kinase in *C*. *albicans* and demonstrates that, similar to *Saccharomyces cerevisiae*, it plays an essential role in signalling for mitotic exit and cytokinesis. Cells depleted of Cdc15 developed into elongated filaments, a common response to cell cycle arrest in *C*. *albicans*. These filaments emerged exclusively from large budded cells, contained two nuclear bodies and exhibited a hyper-extended spindle, all characteristic of these cells failing to exit mitosis. Furthermore these filaments displayed a clear cytokinesis defect, and *CDC15* over-expression led to aberrant cell separation following hyphal morphogenesis. Together, these results are consistent with Cdc15 playing an essential role in signalling for mitotic exit, cytokinesis and cell separation in *C*. *albicans*.

## Introduction

Exit from mitosis following the successful segregation of chromosomes requires the inactivation of the cyclin-dependent kinase (Cdk), and is followed by the activation of cytokinesis and preparation for the onset of the next cell cycle. In budding yeast *Saccharomyces cerevisiae* exit from mitosis and the activation of cytokinesis is regulated by the Mitotic Exit Network (MEN), a GTPase regulated kinase cascade that displays similarity to the fission yeast Septation Initiation Network (SIN) and Metazoan Hippo pathway^1–3^. The core elements of this pathway in budding yeast include the GTPase Tem1, a two-component GTPase activating protein Bub2-Bfa1 and potential GTPase exchange factor Lte1, and the downstream kinases Cdc15 and Dbf2-Mob1 which ultimately regulate the activity of the Cdc14 phosphatase.

In budding yeast Cdc14 is held inactive for the majority of the cell cycle through its interaction with Net1 which sequesters it to the nucleolus. The key output of the MEN is the sustained release of Cdc14 from the nucleolus, which then drives mitotic exit through terminating Cdk activity and activating expression of the Cdk inhibitor Sic1^4–7^. During early anaphase Cdc14 is initially transiently released from the nucleolus to the nucleus through the action of the Cdc14 early anaphase release (FEAR) network^8^. Subsequently, in late anaphase, the MEN then drives the sustained release of Cdc14 into the cytoplasm where it associates with the spindle pole bodies (SPB) and bud neck and drives mitotic exit^4–6,9,10^. The regulation of the MEN is mainly through the spatial and temporal regulation of the GTPase Tem1, which localises to SPB alongside the GAP complex Bub2-Bfa1 which holds it inactive^11–13^. The activity of the GAP complex is maintained by the protein kinase Kin4, which localises to the mother cell cortex and mother cell localised SPB^14–16^. Conversely, Lte1 localises to the daughter cell cortex and is thought to activate Tem1 through preventing the low levels of Kin4 present in the bud from associating with the SPB^17–20^. Therefore the MEN is controlled through spindle position, with Tem1 being activated following the migration of the SPB into the bud. Following its activation Tem1 then signals through the Ste20-like kinase Cdc15 and LATS/NDR kinase Dbf2. GTP-bound Tem1 first activates Cdc15 through its recruitment to the SPB^21,22^, which in turn recruits the Dbf2-Mob1 complex through the phosphorylation of the SPB scaffold protein Nud1^23^. Ultimately, the Dbf2-Mob1 complex then drives the sustained release of Cdc14 to the cytoplasm through its phosphorylation^24,25^.

In addition to signalling mitotic exit more recent work has also suggested the MEN plays a direct role in signalling for cytokinesis and cell separation^26,27^. Firstly, following the drop in Cdk activity, a number of the MEN components have been seen to relocalise to the bud neck. Furthermore, MEN mutants that bypass their mitotic exit defect subsequently exhibit defects associated with actomyosin ring dynamics^28–31^. There is also evidence that activation of Cdc14 results in the repolarisation of the cytoskeleton to the bud neck through impacting on the phosphorylation status of the formins^32^, and that MEN components may also regulate other cytokinesis factors such as Chs2, Hof1 and Inn1^27^. Finally, Cdc14 may also play a role in promoting cell separation through its interaction with the RAM (regulation of Ace2 and morphogenesis) signalling cascade which regulates the enzymes required for septum degradation and cell separation^33^. The finding that the MEN is involved with cytokinesis in budding yeast is also in keeping with the role of analogous pathways in other fungi, such as the fission yeast SIN, where their primary role is in coordinating cytokinesis. Furthermore, a recent re-evaluation of the role of Cdc14 in budding yeast suggests that coordinating cytokinesis is its most conserved function, and that its role in signalling mitotic exit may be through acting on a small set of substrates with additional phosphatases required for widespread Cdk substrate dephosphorylation^7^.

*Candida albicans* is an important opportunistic human fungal pathogen, capable of causing both superficial and life-threatening systemic infections. It can grow in a wide variety of morphological forms, and the ability to switch forms has been linked with virulence. As the process of morphogenesis impacts on the extent of polarised growth, nuclear dynamics, and septation and cell separation its progression must be tightly linked with cell cycle control^34^. A number of key constituents of the MEN have now been studied in *C*. *albicans* including Tem1, Dbf2 and Cdc14^35–37^. However, strains depleted of these components demonstrate a range of different phenotypes, suggesting that in *C*. *albicans* this network is not a simple linear signalling cascade that operates through the release and activation of Cdc14 as a common end point. The GTPase Tem1 is essential, with cells depleted of it forming filaments and exhibiting a clear mitotic exit defect through arresting with an extended spindle^37^. In contrast, although the downstream kinase Dbf2 was also shown to be essential its primary function was seen to be in regulating spindle organisation and signalling for actomyosin ring contraction^36^. Finally, Cdc14 is not essential in *C*. *albicans* and mainly acts to drive cell separation following cytokinesis, through its interaction with the RAM pathway impacting on the localisation of the Ace2 transcription factor^35^. This work characterises the role of the final key component in the MEN signalling cascade, the Ste20-like kinase Cdc15. *CDC15* is seen to be essential in *C*. *albicans*, and cells depleted of Cdc15 display phenotypes highly reminiscent of Tem1 depleted cells. Following Cdc15 depletion the cells formed filaments that emerged exclusively from large budded cells. However, these filaments ultimately lost viability and displayed clear evidence of a mitotic exit and cytokinesis defect, with the depleted cells arresting with a hyper-extended spindle, and failing to undergo cytokinesis and enter subsequent rounds of nuclear division. These phenotypes are therefore consistent with *CDC15* playing an essential role in signalling for mitotic exit and cytokinesis.

## Results

### Identification and localisation of *C*. *albicans* Cdc15

The *C*. *albicans* genome contains a single open reading frame (orf19.3545, C2_05140W_A) which displays extensive homology to *S*. *cerevisiae* Cdc15, and whose overexpression we have previously demonstrated rescues the essential phenotype associated with the loss of Tem1 in *C*. *albicans*^37^. This *CDC15* gene encodes a putative 1126 amino acid protein, which displays 20.8% identity (38.4% similarity) across the length of the protein to *Sc*Cdc15. It is however slightly larger than its *S*. *cerevisiae* homolog, of 974 amino acids, due to the presence of an extended N terminal region. As expected it contains all the protein motifs associated with it being a serine and threonine protein kinase (residues 211–460), plus it also displays a potential armadillo like fold in the C terminal region which may play a role in protein-protein interactions.

**Figure 1:**
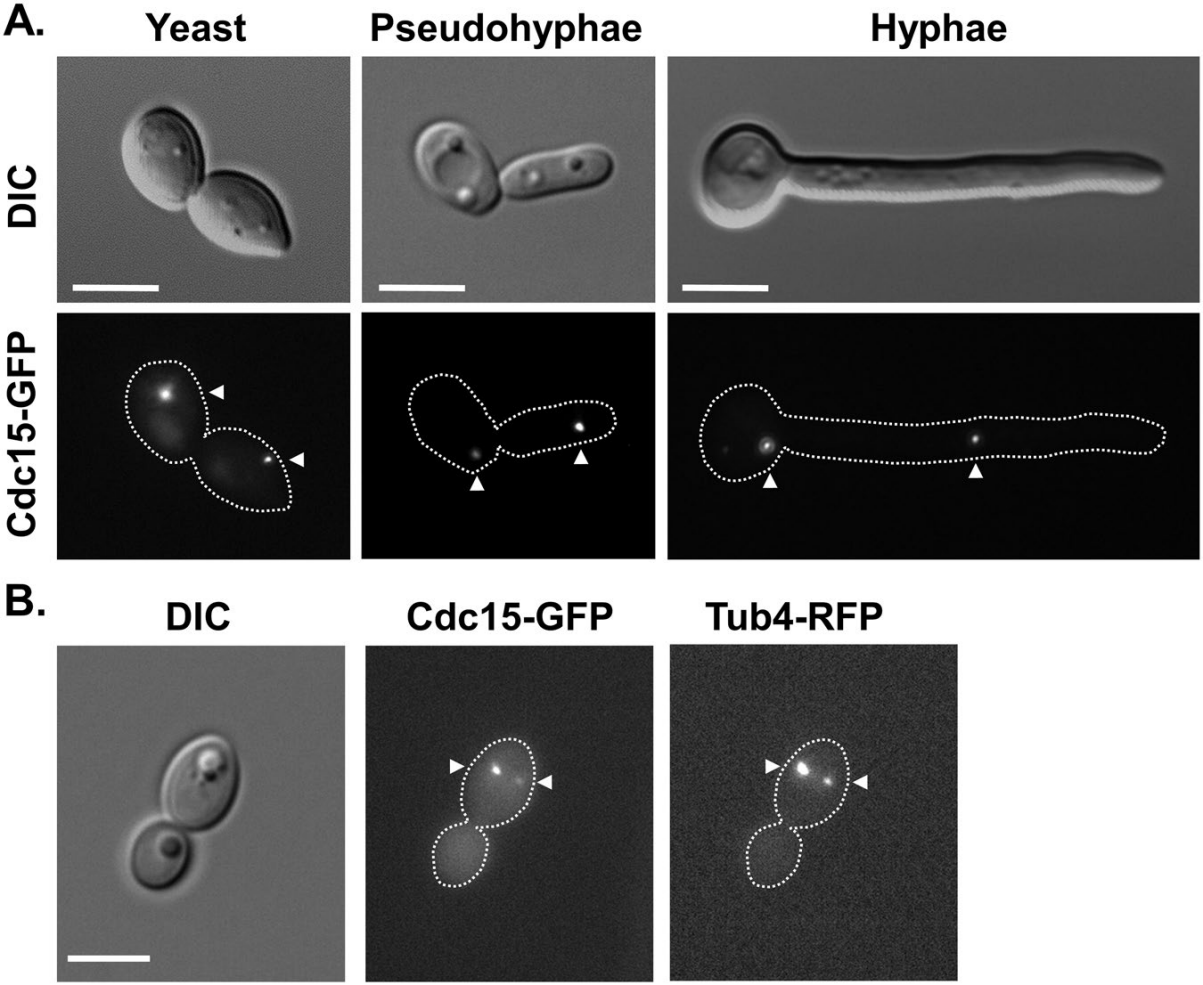
Cdc15-GFP localises to spindle pole bodies in *C*. *albicans*. (**A**) Cdc15-GFP, under control of the *CDC15* native promoter, was visualised in *C*. *albicans* grown as yeast (YEPD, 30 °C), pseudohyphae (YEPD, 37 °C) or true hyphae (YEPD + 20% Serum, 37 °C). (**B**) Cdc15-GFP was demonstrated to co-localize with RFP tagged gamma-tubulin (Tub4-RFP). Differential interference contrast (DIC) and fluorescent images were taken of unfixed cells, and arrows indicate the position of Cdc15-GFP and Tub4-RFP. Bars, 5 µm.

In order to follow the *in vivo* localisation of Cdc15 in *C*. *albicans* strains were constructed expressing a C-terminally tagged GFP fusion protein, Cdc15-GFP, from either the native *CDC15* promoter or the more highly expressed tetracycline-regulated promoter. No change in the localisation pattern of Cdc15-GFP was seen following over-expression. In *S*. *cerevisiae* Cdc15 is predominantly found associated with the spindle pole body; and in keeping with this the Cdc15-GFP fusion protein was also seen to localise to one or two intense spots in *C*. *albicans* yeast, psuedohyphal and hyphal cells (Fig. 1A). To confirm this spindle pole body localisation the gamma-tubulin gene, *TUB4*, was tagged with RFP in the *CDC15*-*GFP* background. This strain clearly demonstrated the co-localisation of Cdc15-GFP and Tub4-RFP (Fig. 1B), therefore confirming the localisation of Cdc15 on the spindle pole body. This localisation was also seen to be cell cycle regulated, similar to what we have previously reported for Tem1^37^. From scoring localisation in an asynchronous culture of yeast cells the majority of unbudded cells (58%, n = 96), which would be in the G1 phase of the cell cycle, displayed no Cdc15-GFP signal. However, 70% (n = 60) of small budded cells, characteristic of the G1/S phase transition, contained one distinct Cdc15-GFP spot. Furthermore, at later stages of the cell cycle 84% (n = 56) of large budded cells were seen to contain two spots following the duplication of the spindle pole body. Cdc15 therefore follows a similar localisation pattern to Tem1 in *C*. *albicans*, becoming localised to the spindle pole body commensurate with the onset of the S-phase of the cell cycle. In addition to its spindle pole body localisation *S*. *cerevisiae* Cdc15 has also been reported to localise to the bud neck late in the cell cycle^38^, and in *C*. *albicans* Dbf2 and Cdc14 have also been seen to localise to the bud neck late in the cell cycle^35,36^. However, similar to what was previously seen with Tem1^37^, there was no evidence of Cdc15-GFP re-localising to the bud neck even when overexpressed.

### *CDC15* is essential in *C*. *albicans*

**Figure 2:**
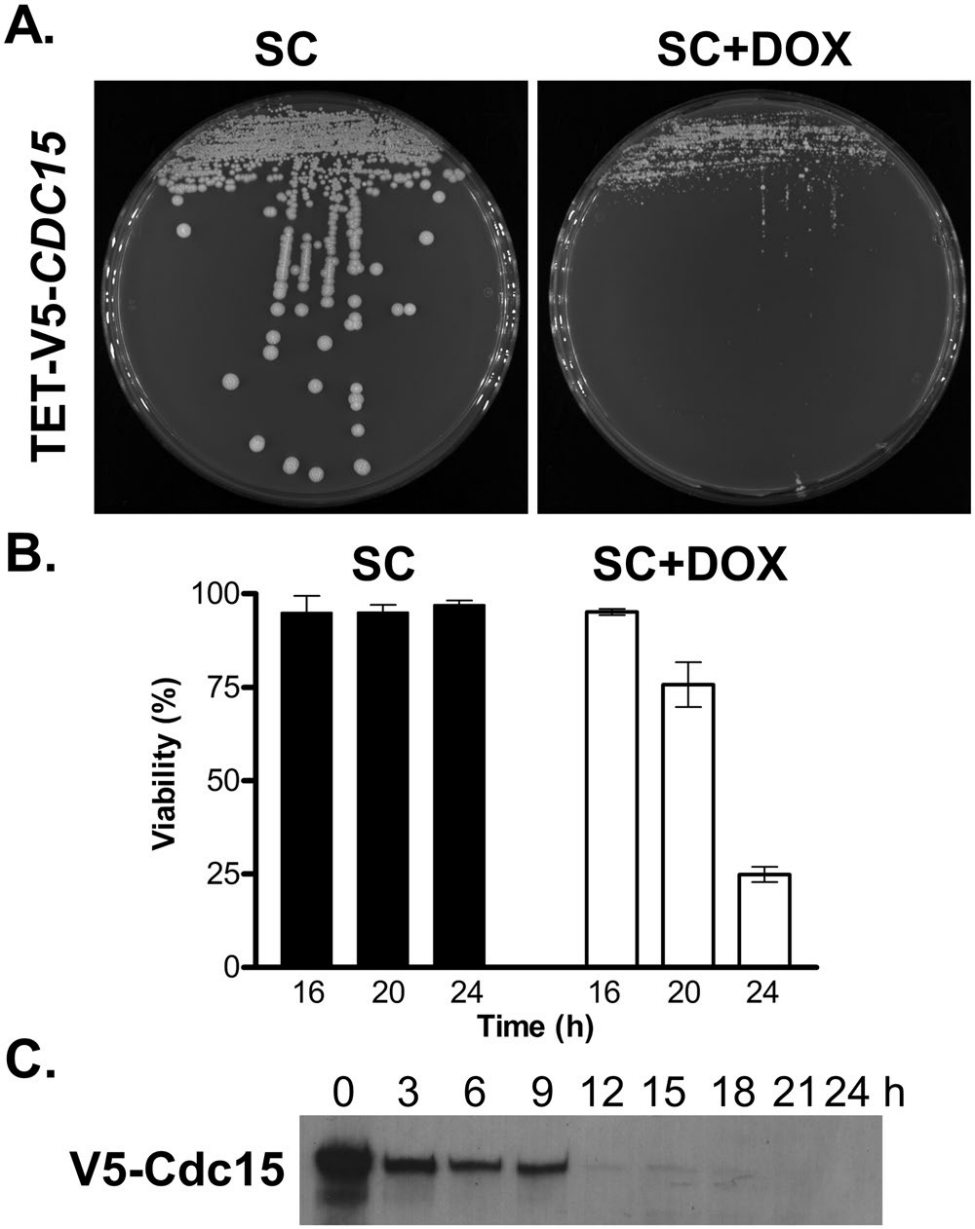
*CDC15* is essential in *C*. *albicans*. (**A**) Growth of *C*. *albicans* TET-V5-CDC15 on SC medium (*CDC15* expression on) and SC medium plus 20 µg/ml doxycycline (SC + Dox, *CDC15* expression off). (**B**) Viability of Cdc15 depleted cells. The TET-V5-CDC15 strain was grown from 2.5 × 10^3^ cells/ml under non-repressing (SC) or repressing conditions (SC + Dox) and viability determined at the time points indicated. (**C**) V5-Cdc15 protein levels following promoter shut off. TET-V5-CDC15 was grown under repressing conditions for the times indicated and V5-Cdc15 protein levels determined by western blotting.

In order to study the function of Cdc15 a strain carrying a single copy of *CDC15* under the control of the tetracycline repressible promoter was generated (SBC189, TET-CDC15). In addition this system was also modified to incorporate an N-terminal V5 tag, resulting in strain TET-V5-CDC15 (SBC190), allowing the levels of Cdc15 to be monitored. The TET-CDC15 and TET-V5-CDC15 strains grew normally under non-repressing conditions and formed colonies. In contrast, under repressing conditions, through the addition of 20 µg/ml doxycycline, these strains failed to form true colonies (Fig. 2A). Some microcolonies were seen to form under repressing conditions, but these were abortive and failed to develop further or be successfully subcultured under repressing conditions, suggestive of *CDC15* being essential in *C*. *albicans*. To further confirm the essential nature of *CDC15* the single transformation gene function test, using the *UAU1* (*ura3*-*ARG4*-*ura3*) cassette^39^, was also employed. Screening of sixty arginine and uridine prototrophic recombinants derived from a *CDC15*/*cdc15*::*UAU1* strain demonstrated all had undergone a triplication event and carried a wild type copy of *CDC15*, consistent with *CDC15* being essential. Furthermore, cells depleted of Cdc15 were ultimately seen to lose viability. In liquid culture this was first seen following 20 h growth of the TET-V5-CDC15 strain under repressing conditions with a significant drop in viability to 75.77 ± 5.99% (p < 0.001), and subsequently to 24.91 ± 2.06% (p < 0.0001) at 24 h, as opposed to under non-repressing conditions where viability was maintained at over 95% (Fig. 2B). These results are in contrast to a previous large scale screen of kinases in *C*. *albicans* where insertional mutants in *CDC15* were reported^40^. However, the two mutants previously reported would have only affected the extreme C-terminus of the protein, resulting in a slight truncation (deletion of either the C terminal 86 or 125 Aa of the 1126 Aa protein) and would not have impacted on the kinase domain. The results presented here therefore clearly demonstrate that *CDC15* is essential in *C*. *albicans*.

### Cells depleted of Cdc15 form elongated filaments

**Figure 3:**
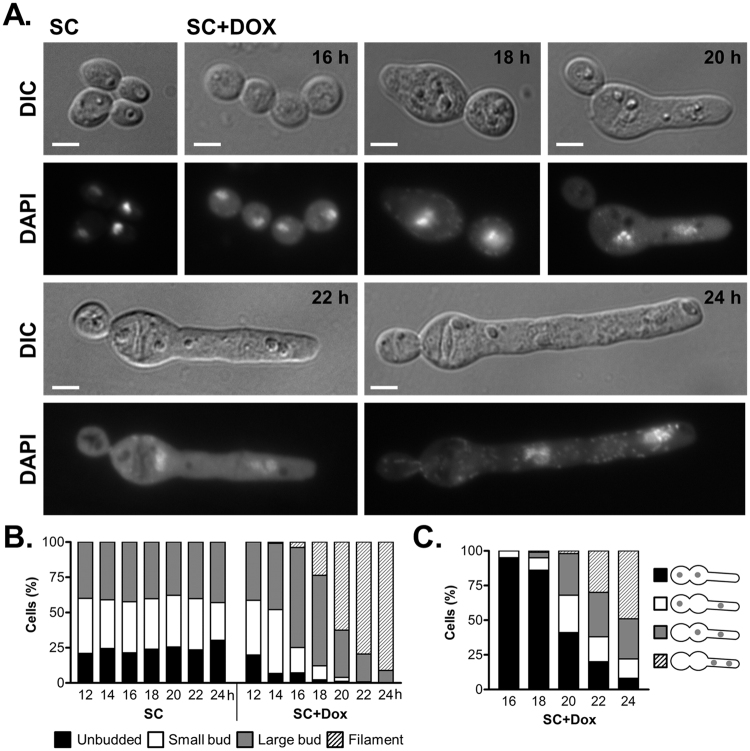
Cdc15 depleted cells form filaments and fail to exit mitosis. (**A**) The TET-V5-CDC15 strain was grown, from 2.5 × 10^3^ cells/ml, under non-repressing (SC) or repressing conditions (SC + Dox) for the times indicated, fixed and stained with DAPI. Representative cells are shown. Bars 5 µm. The TET-V5-CDC15 cultures were also scored for cellular morphology (**B**) and nuclear position (**C**), with at least 100 cells counted for each time point. (**B**) For cellular morphology cells were classed as either unbudded, small budded, large budded or large budded with a filament emerging. (**C**) The binucleate large budded cells and large budded cells with filaments emerging were scored according to nuclear position with: one nucleus in original mother and daughter cell, one nucleus in mother and one in filament, one nucleus in daughter and one in filament, and both nuclei in the filament.

To characterise the terminal phenotype associated with loss of *CDC15* cellular morphology was monitored following the repression of *CDC15* expression in the TET-V5-CDC15 strain. Following overnight growth in non-repressing conditions the TET-V5-CDC15 strain was inoculated into repressing conditions (SC + Dox) at 2.5 × 10^3^ cells/ml, and cells collected at various time points. Under these conditions the cells grew normally for the first 12 h before starting to arrest as large budded cells (Fig. 3A,B), such that by 16 h 71.01 ± 5.37% of the population was present as large budded cells compared to 42.38 ± 1.9% in an exponential culture under non-repressing conditions (SC). Western blot analysis of the TET-V5-CDC15 strain grown under repressing conditions also demonstrated that V5-Cdc15 levels were almost undetectable following 12 h growth (Fig. 2C), therefore the arrest seen broadly correlated with the depletion of Cdc15. Following their arrest as large budded cells the Cdc15 depleted cells subsequently became enlarged and initiated filamentous growth. In terms of cell size, under the conditions tested, non-repressed cells demonstrated an average width of 4.37 ± 0.32 µm (n = 53), however, between 16 and 20 h the cells were seen to swell from a width of 4.14 ± 0.55 µm (n = 50) at 16 h to 6.57 ± 1.25 µm (n = 66) at 20 h. From 16 h these cells also started to demonstrate filamentous growth, with filaments emerging exclusively from large budded cells, such that by 24 h the majority of cells in the population (91.13 ± 1.40%) displayed filamentous growth. Growth of the TET-V5-CDC15 strain, under non-repressing conditions for yeast like growth, displayed no obvious phenotype, indicating that the arrest followed by cell swelling and initiation of filamentous growth was due to the loss of *CDC15*.

### Cdc15 is required for mitotic exit and cytokinesis

Our previous work has shown that Tem1 is required for mitotic exit and cytokinesis, and suggested that its essential function is signalled through Cdc15^37^. To confirm that depletion of Cdc15 also results in nuclear division defects the TET-V5-CDC15 cells were collected following repression and nuclear material visualised through staining with DAPI. Following 16 h repression, when the majority of cells were arrested, over 90% of these cells were binucleate, with a small proportion of multinucleate cells. Furthermore, at all subsequent time points this was maintained, with over 90% of cells remaining binucleate (Fig. 3). Following the formation of filaments by the repressed cells nuclear migration was also seen. Initially one nucleus from the original budded cell body was seen to migrate into the filament, and it was subsequently followed by the second. As a result, following 24 h repression, both nuclei were present in the filaments of 50% of the arrested cells (Fig. 3A,C).

**Figure 4:**
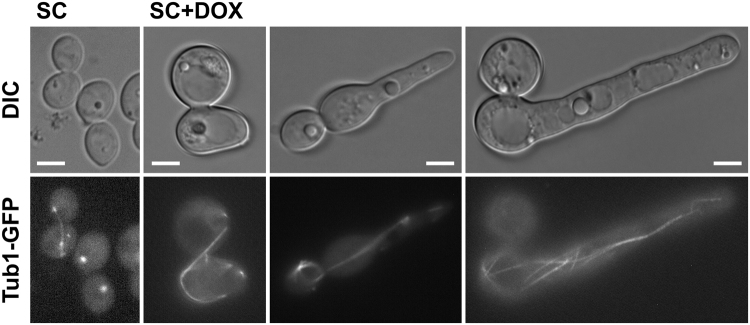
Cdc15 depleted cells display a hyper-extended mitotic spindle. Tub1-GFP was visualised in the TET-V5-CDC15 strain background after 16 h growth in non-repressing (SC) or repressing (SC + Dox) conditions. Cells representative of the various morphologies displayed following Cdc15 depletion are shown. Bars, 5 µm.

The nuclear dynamics seen following Cdc15 depletion are in keeping with these cells displaying a defect in mitotic exit and failing to re-enter the cell cycle. To confirm this mitotic exit defect spindles were visualised through GFP tagging Tub1 in the *CDC15* tetracycline repressible strains. Under repressing conditions these strains were also seen to arrest as large budded cells which subsequently formed filaments. However, the GFP tagging of Tub1, in both the TET-CDC15 and TET-V5-CDC15 strains, resulted in the cells showing much less synchrony in their response to Cdc15 depletion. The cells did all arrest, generally earlier than the untagged strain, but a greater level of variation was seen in the timing of this. As such at time points following *CDC15* repression the cell population would demonstrate a range of morphologies associated with Cdc15 depletion. Importantly however, the vast majority of these arrested cells displayed an extended spindle (Fig. 4), characteristic of the cells arresting in late anaphase or early telophase and failing to exit from mitosis. The mitotic spindle in these cells was also seen to become highly elongated, displaying a fish hook or lasso morphology, with the spindle wrapped along the cell periphery. A similar phenotype has been reported in a subset of *S*. *cerevisiae* mutants, including a number in the MEN^41–44^. Thus Cdc15 depleted cells display both a defect in mitotic exit and the regulation of spindle length.

**Figure 5:**
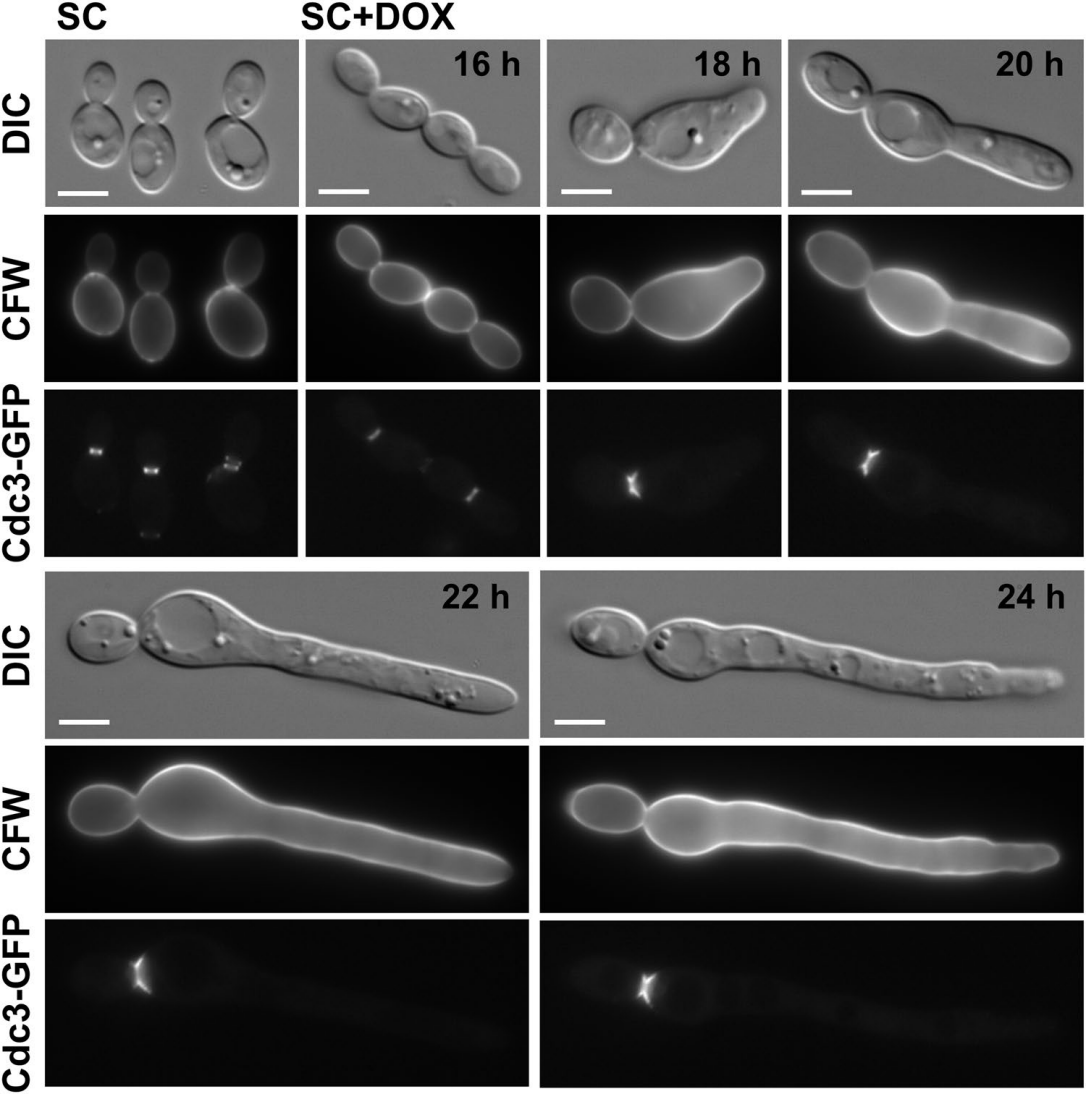
Cdc15 depleted cells fail to septate. The TET-V5-CDC15 strain, expressing the septin Cdc3 tagged with GFP, was grown in non-repressing (SC) or repressing conditions (SC + Dox) for the times indicated. DIC, calcofluor white (CFW) and Cdc3-GFP images of representative cells are shown. Bars, 5 µm.

The presence of a hyper-extended spindle and nuclear migration into the filaments, following Cdc15 depletion, is suggestive of cytokinesis being blocked in these cells. To confirm this the Cdc15 depleted cells were first stained with calcofluor white (CFW), and the arrested cells were seen to display only a single chitin band at the original mother-bud neck with no bands present in the filaments (Fig. 5). In order to follow this further the septin Cdc3 was GFP tagged in the TET-V5-CDC15 strain. In wild type cells a septin ring is laid down early in the cell cycle at the site of bud emergence, and this then switches to an hourglass-shaped collar across the mother-bud neck before finally splitting into two rings for cytokinesis^45,46^. Following Cdc15 depletion Cdc3-GFP was only seen at the original mother-bud neck of the arrested cells, with no structures present in the filaments (Fig. 5). Furthermore, the septins in these cells all displayed the hourglass collar structure, consistent with the cells having arrested before the signal for the septin rings to separate and the actomyosin ring to assemble. Cdc15 depleted cells are therefore one continuous compartment and have a clear cytokinesis defect.

**Figure 6:**
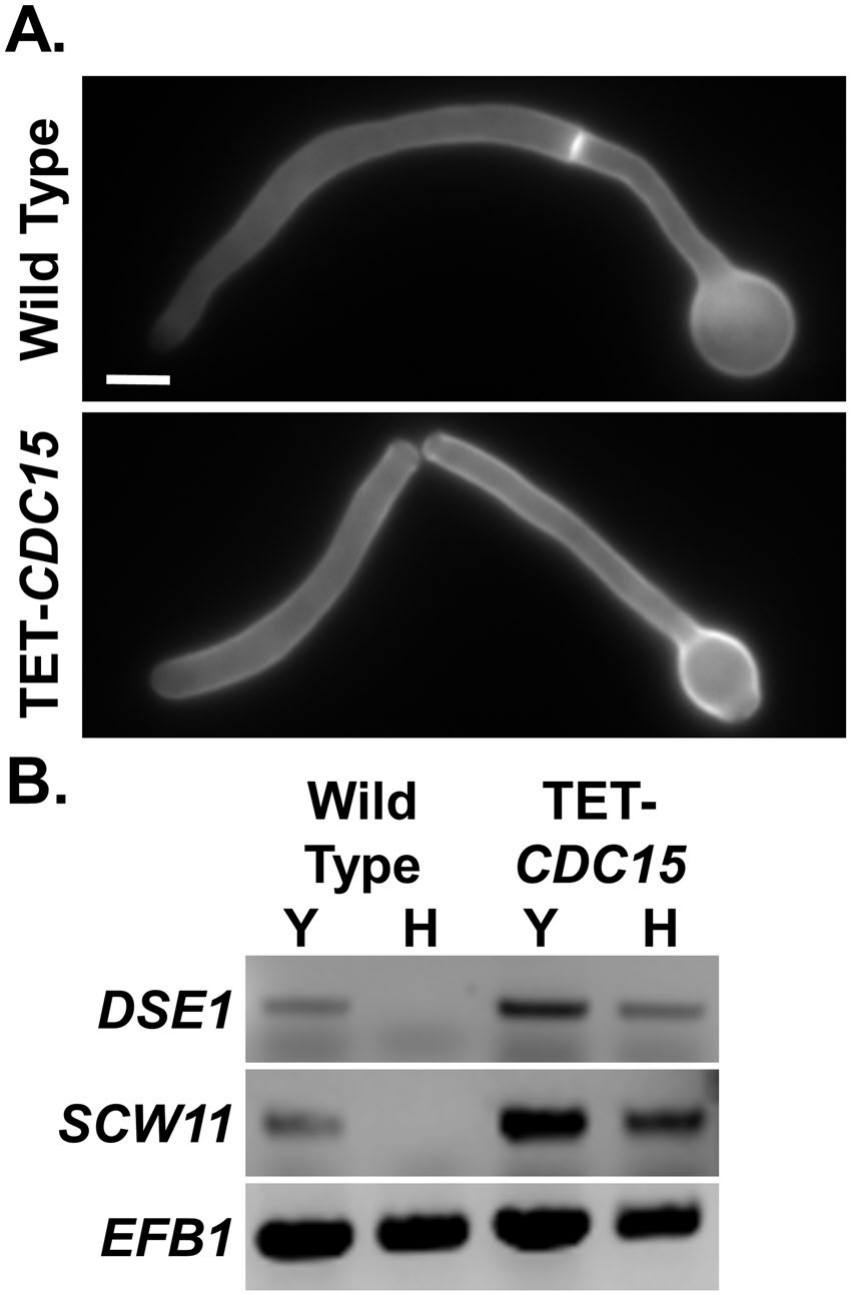
Hyphae overexpressing *CDC15* activate cell separation. (**A**) Wild type (NGY152) and the TET-CDC15 strain, which overexpresses *CDC15*, were grown for 3 h in hyphal inducing conditions, fixed, and stained with CFW. Bar, 5 µm. (**B**) Expression analysis of Ace2-regulated genes *DSE1* and *SCW11* in yeast form (Y) or hyphal form (H), *EFB1* was used as a loading control.

Finally, evidence that Cdc15 also plays a role in signalling for cell separation was seen through following hyphal growth in the TET-CDC15 strain, which under non-repressing conditions overexpresses *CDC15*. This strain grew normally under non-repressing conditions that promote yeast growth, and it also switched to hyphal growth as expected in response to serum and temperature. However, in contrast to wild type cells, where cell separation is blocked following filamentation, the filaments formed under non-repressing conditions in response to serum by the TET-CDC15 strain displayed partial or total separation (Fig. 6A). The Ace2 transcription factor in known to drive expression of genes involved with cell separation, such as *DSE1* and *SCW11*^35,47^. Expression analysis clearly demonstrated that these genes were repressed as expected in wild type hyphae, however the TET-CDC15 strain still displayed their expression when grown in hyphal form (Fig. 6B). A similar phenotype has been reported in a *sep7* mutant which lacks hyphal specific modifications to the septin ring, and mutants in *mob2* which is part of the RAM network involved in regulating Ace2^48,49^. This would suggest that the MEN is involved with activating Ace2 and signalling for cell separation, however this signal must normally be overridden in hyphal cells. Overall the phenotypes associated with *CDC15* are therefore consistent with it playing a key role in the MEN and signalling for mitotic exit, cytokinesis and cell separation.

## Discussion

This report focuses on the role of Cdc15 in the processes of mitotic exit, cytokinesis and cell separation in *C*. *albicans*. Cdc15 was seen to be essential in *C*. *albicans* and cells depleted of it displayed phenotypes highly similar to cells depleted of Tem1. This, together with the previous finding that overexpression of *CDC15* rescues the essential phenotype associated with the loss of Tem1^37^, is in keeping with Cdc15 signalling downstream of Tem1 and playing an essential role in mitotic exit. The essentiality of *CDC15* was demonstrated through two approaches, plus further supported by the loss of viability seen following Cdc15 depletion. It was however noticeable that when using the tetracycline repressible promoter system the resulting strain completed multiple rounds of cell division before arrest was seen. This would suggest that Cdc15 is a stable protein and only low levels are required for its essential function. A similar result was seen following Tem1 depletion^37^, plus the essentiality of Dbf2 following its depletion was only observed when coupled with epitope tagging^36^. This observation, that only low levels of MEN components are required for viability, is also supported by a recent re-evaluation of the role of Cdc14 in *S*. *cerevisiae* which suggested that only trace levels of the protein are required for signalling mitotic exit^7^.

When cells were depleted of Cdc15 they formed wide filamentous structures, typically lacking constrictions. The activation of polarised growth by *C*. *albicans* is a common response to cell cycle arrest, and is seen to occur independent of the stage of the cell cycle the cells have arrested in^34^. However, the morphology seen in the progenitor cell giving rise to the filament will vary depending on the stage of arrest. In the case of the Cdc15 depleted cells, similar to what was previously reported with cells depleted of Tem1 or the G2/M cyclin Clb2^37,50^, the filaments appeared exclusively from large budded cells. This phenotype is therefore consistent with Cdc15 depleted cells displaying a defect late in the cell cycle. Furthermore, nuclear dynamics and the presence of a hyper extended spindle are indicative of Cdc15 depleted cells failing to exit mitosis. The filamentous cells formed predominantly contained two nuclear bodies, indicating that Cdc15 depleted cells were arrested after nuclear division. These cells also displayed an extended spindle, indicative of the cells arresting in either late anaphase or early telophase and failing to exit mitosis. Indeed the spindle in these cells became hyper-extended and was wrapped around the cell periphery, which would suggest that the inactivation of mitotic cyclins is required to promote spindle disassembly. A similar phenotype has been seen in specific groups of *S*. *cerevisiae* mutants, in particular members of the CFT19 complex which associates with the kinetochore, the FEAR network, and importantly the MEN^41–44^.

In addition to the mitotic exit defect there was also evidence for a cytokinesis defect in Cdc15 depleted cells, as demonstrated by the lack of septa being laid down along the filaments. There was some evidence of a chitin band at the original mother-bud neck, however, the migration of nuclei across the neck and ultimately into the filaments is supportive of the view that these cells consist of one continuous compartment. Furthermore, septins were also only present at the original mother-bud neck in the arrested cells, and were limited to their hourglass collar structure failing to separate into two rings. A similar phenotype was seen in *S*. *cerevisiae*, and following Tem1 depletion in *C*. *albicans*, and is supportive of the view that the MEN also acts in signalling for cytokinesis. Evidence was also seen supporting the view that the MEN promotes cell separation. When *C*. *albicans* forms hyphae cell separation is normally blocked, however, the overexpression of *CDC15* overcame this and resulted in cell separation. Previous work has shown that Cdc14 plays a role in promoting cell separation, and that this is blocked in hyphae through the action of the hyphal specific cyclin Hgc1 which prevents Cdc14 localising to the site of septation^35^. The overexpression of Cdc15 may potentially be outcompeting the signal from the Hgc1-Cdk complex and driving localisation of Cdc14 to the septum region. This could be achieved either though Cdc15 directly driving its activation and localisation or potentially through impacting on the hyphal specific septin (Sep7) which has been seen to block Cdc14 recruitment^48^. Either way this overexpression ultimately led to the aberrant activation of the RAM signalling cascade in hyphae which then drove expression of genes required for cell separation. Cross talk between the MEN and RAM signalling cascade has been reported in *S*. *cerevisiae*^33^, and this work alongside previous reports would suggest that this also occurs in *C*. *albicans* yeast cells but this signal is overridden in hyphal cells to block cell separation.

Cdc15 was seen to localise to the SPB, and this localisation was cell cycle regulated. Similar to the findings in *S*. *cerevisiae*^21,22^, and for Tem1 in *C*. *albicans*^37^, Cdc15 localised to the SPB commensurate with the G1/S phase transition. In *S*. *cerevisiae* Cdc15 has also been reported to relocalise to the bud neck at the end of mitosis^51^, as have Dbf2 and Cdc14 in *C*. *albicans*^35,36^. However, no evidence was seen of Cdc15 relocalising to the bud neck in *C*. *albicans*. It instead remained exclusively localised to the SPB, similar to the localisation pattern previously reported for Tem1. A similar pattern of localisation exclusive to the SPB has also been seen for the Cdc15 homolog (Sid1) in *S*. *pombe*, although here localisation was asymmetrical and only seen on one SPB^52^. Overall the localization of Cdc15 in *C*. *albicans* would suggest that its role in cytokinesis and cell separation must therefore be signalled through other factors, such as Dbf2 and Cdc14.

**Figure 7:**
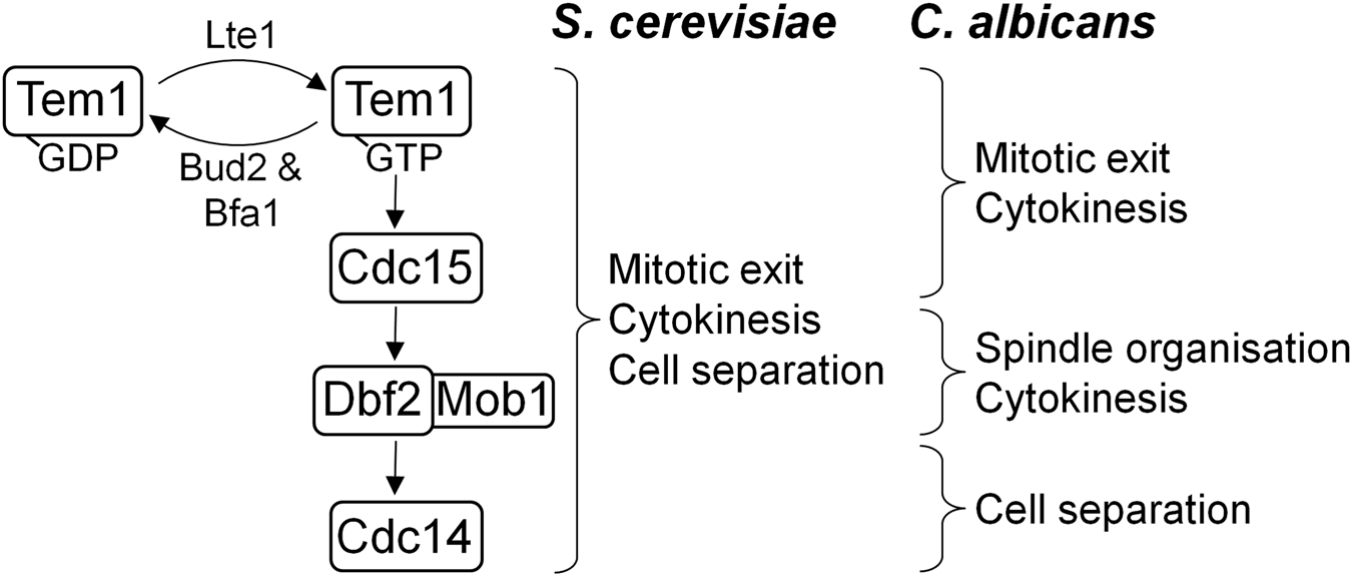
Key roles of MEN components in *S*. *cerevisiae* and *C*. *albicans*.

This report, alongside the previous analysis of *C*. *albicans* MEN components^35–37^, is supportive of the network playing a key role in driving mitotic exit, cytokinesis and cell separation similar to the situation in *S*. *cerevisiae*. However, although the overall role of the network is conserved, there are key differences in the role of the individual components of the network in these two yeasts (Fig. 7). In *S*. *cerevisiae* the key signalling components of the network, Tem1, Cdc15, Dbf2/Dbf20 and Cdc14, are all essential and signal for mitotic exit through the release and activation of Cdc14, following which they act to drive cytokinesis and cell separation^1–3,5,26,27^. As such mutants in these components all share a common phenotype, arresting as large budded cells with an extended spindle. In contrast cells depleted of the MEN components in *C*. *albicans* display a range of phenotypes. Cells depleted of either Tem1^37^ or Cdc15 arrest after nuclear division with an elongated spindle, consistent with these factors signalling for mitotic exit. In contrast previous reports suggest the primary role of Dbf2 is in signalling cytokinesis^36^, whereas the role of the non-essential Cdc14 is in driving cell separation^35^. Therefore, although the MEN is conserved between *C*. *albicans* and *S*. *cerevisiae*, and Tem1 and Cdc15 signal for mitotic exit in both species, the final output from the network that drives mitotic exit must differ. Whereas the network signals for mitotic exit through Cdc14 in *S*. *cerevisiae*, in *C*. *albicans* this must be achieved through other factors downstream of Cdc15, perhaps through the PP1 and PP2A phosphatases as seen in other systems^53,54^. Indeed the initial analysis of PP1 and PP2A components in *C*. *albicans* has provided some evidence of them playing an essential role in late cell cycle progression^55,56^.

## Methods

### Strains, media and growth conditions

All strains used and constructed in this study are listed in Table 1. *C*. *albicans* strains were grown at 30 °C in YEPD (1% yeast extract, 2% peptone, 2% glucose) or in SC (0.67% yeast nitrogen base with ammonium sulphate without amino acids, 2% glucose, 0.077% complete supplement mixture minus uracil), supplemented with 50 µg/ml uridine as required. Nourseothricin resistance was selected for through growth on Sabouraud dextrose agar (1% mycological peptone, 4% glucose, 1.5% agar) containing 200 µg/ml nourseothricin (Werner BioAgents). Hyphal growth was induced by growing the cells at 37 °C in YEPD medium plus 20% fetal calf serum. To repress expression from the tetracycline-regulated promoter doxycycline was added to the media at a final concentration of 20 µg/ml. To score for viability the cells were first washed in PBS and the total cell number established through a haemocytometer count, the proportion of viable cells was then determined following the plating of serial dilutions onto YEPD-agar medium.

**Table 1:** Strains used in this study.

Strain	Genotype	Reference
CAI-4	*ura3*Δ::*imm434*/*ura3*Δ::*imm434*	^63^
THE1	*ura3*Δ::*imm434*/*ura3*Δ::*imm434 ade2*Δ::*hisG*/*ade2*Δ::*hisG ENO1*/*eno1*::*ENO1-tetR-ScHAP4AD-3xHA-ADE2*	^57^
SN148	*arg4*Δ::*dpl200*/*arg4*Δ::*dpl200*, *leu2*Δ::*dpl200*/*leu2*Δ::*dpl200*, *his1*Δ::*hisG*/*his1*Δ::*hisG*, *ura3*Δ::*imm434*/*ura3*Δ::*imm434*	^64^
NGY152	As CAI-4 but *RPS1*/*rps1*Δ::CIp10	^65^
SBC187	As THE1 but *CDC15*/*cdc15*Δ::*dpl200-URA3-dpl200*	This study
SBC188	As THE1 but *CDC15*//*cdc15*Δ::*dpl200*	This study
SBC189	As THE1 but *URA3-TETp-CDC15*/*cdc15*Δ::*dpl200*	This study
SBC190	As THE1 but *URA3-V5-TETp-CDC15*/*cdc15*Δ::*dpl200*	This study
SBC191	As THE1 but *URA3-V5-TETp-CDC15*/*cdc15*Δ::*dpl200*, *TUB1*/*TUB1-GFP-NAT1*	This study
SBC192	As NGY152 but *CDC15*/*CDC15*-*GFP*-*NAT1*	This study
SBC193	As THE1 but *CDC15*/*URA3*-*TETp*-*CDC15*	This study
SBC194	As THE1 but *CDC15*/*URA3*-*TETp*-*CDC15*-*GFP*-*NAT1*	This study
SBC195	As THE1 but *CDC15*/*ura3*-*TETp*-*CDC15*-*GFP*-*NAT1*	This study
SBC196	As THE1 but *CDC15*/*ura3*-*TETp*-*CDC15*-*GFP*-*NAT1*, *TUB4*/*TUB4*-*RFP*-*URA3*	This study
SBC197	As SN148 but *CDC15*/*cdc15*::*UAU1*	This study
SBC198	As THE1 but *URA3-V5-TETp-CDC15*/*cdc15*Δ::*dpl200*, *CDC3*/*CDC3-GFP-NAT1*	This study

### Strain construction

The strains constructed in this study (Table 1) were generated through a targeted recombination approach using PCR-generated disruption or tagging cassettes, and the oligonucleotides used are listed in Table S1. To regulate expression of *CDC15* a conditional mutant was generated that carries a single copy of *CDC15* under the control of a tetracycline-regulated promoter^57^. For this the first copy of *CDC15* was disrupted using the *URA3* recyclable PCR-directed disruption system^58^ in the THE1 strain which expresses the tetracycline transactivator^57^. The disruption cassette was first amplified from pDDB57 using the primer pair CDC15-KO-F and CDC15-KO-R and transformed into the THE1 strain. The *URA3* marker was then recycled through selection for *ura3* auxotrophs on SC medium plus 5-fluoroorotic acid (1 mg/ml) and uridine (50 µg/ml). The promoter replacement cassette was then amplified from p99CAU1^57^ using primer pair CDC15-TET-F and CDC15-TET-R, and transformed into the heterozygote mutant to generate the TET-CDC15 strain (SBC189). In addition, to allow the levels of Cdc15 to be monitored following promoter switch off, the tetracycline-repressible system was modified to include an N-terminal V5 epitope tag. For this the promoter replacement cassette was amplified from p99CAU1 with primer pair TET-F and TET-V5-R and cloned into the pGEM-T Easy vector system (Promega) to generate pURA3-TET-V5. The promoter replacement cassette was then amplified from this vector using primer pair CDC15-TET-F and CDC15-TET-V5-R and transformed into the heterozygote mutant to generate the TET-V5-CDC15 strain (SBC190). *CDC15* was also disrupted using the *UAU1* cassette^39^, for this the cassette was amplified from the plasmid pBME101 using the primer pair CDC15-UAU-F and CDC15-UAU-R and transformed into strain SN148.

For the C- terminal tagging of Tub1 and Cdc3 GFP-NAT1 tagging cassettes were amplified from pGFP-NAT1^59^ using primer pairs TUB1-GFP-F and TUB1-NAT1-R, and CDC3-GFP-F and CDC3-NAT1-R. The resulting cassettes were then transformed into the TET-V5-CDC15 strain with selection for nourseothricin resistance. For localisation studies Cdc15 was C-terminally tagged with GFP, for this a GFP-NAT1 tagging cassette^60^ was amplified using primer pair CDC15-GFP-F and CDC15-NAT1-R and transformed into the NGY152 strain. In addition a strain was also constructed where *CDC15*-*GFP* was overexpressed through replacing the native promoter with the tetracycline- regulated promoter. For this the promoter replacement cassette was first amplified from p99CAU1 using primer pair CDC15-TET-F and CDC15-TET-R and transformed into the THE1 strain, and the resulting strain was then transformed with the *CDC15*-*GFP*-*NAT1* tagging cassette. Finally, for co-localisation studies, Tub4 was RFP tagged in the *CDC15*-*GFP* overexpression background. For this the strain was first rendered auxotrophic for uracil through selection on 5- fluoroorotic acid, and Tub4 was then RFP tagged through transformation with a RFP-URA3 tagging cassette^61^ amplified with primer pair TUB4-RFP-F and TUB4-URA3-R.

### Cell staining and microscopy

For microscopy cell samples were washed in PBS and viewed live for visualising the fluorescent protein fusions and calcofluor white (CFW) staining, or following a 10 min fixation in 70% ethanol for 4,6-diamidino-2-phenylindole dihydrochloride (DAPI) staining. To visualise septa and nuclei cells were stained for 5 min with CFW (5 µg/ml) or DAPI (1 µg/ml) respectively. Epifluorescence and differential interference microscopy was carried out using a Zeiss Axiophot microscope equipped with a Plan Neofluor 100×/1.30 oil objective. Images were captured using a Spot Pursuit™ 1.4 MP Monochrome CCD camera and Visiview software (Visitron Systems). Images were subsequently handled and measurements taken using the Fiji software^62^. Statistical differences were determined using a one way ANOVA with Turkey’s post hoc test.

## Electronic supplementary material

 Supplementary Information
