# Supplementary material for: Candida albicans Cdc15 is essential for mitotic exit and cytokinesis
Source: Sci Rep. 2018 Jun 11;8:8899. doi: 10.1038/s41598-018-27157-y (PMC5995815; doi:10.1038/s41598-018-27157-y)
Supplement: Supplementary file 1 — Supplementary Information [file 41598_2018_27157_MOESM1_ESM.doc]

***Candida albicans* Cdc15 is essential for mitotic exit and cytokinesis**

**Steven Bates**

**Biosciences, College of Life and Environmental Sciences, University of Exeter, Exeter EX4 4QD, UK**

**Correspondence and requests for materials should be addressed to S.B. (email:** [**s.bates@ex.ac.uk**](mailto:s.bates@ex.ac.uk)**)**

Table S1. Oligonucleotides used in this study

| **Primer** | **Sequence (5´ to 3´)^a^** |
| --- | --- |
| CDC15-KO-F | ggttttcttttttaggattttcaactacgtgacattctcttttgttctcaactatgtgcatagaactatagaaatgTGTGGAATTGTGAGCGGATA |
| CDC15-KO-R | cgttgcttctctttgtttttttgctactattataaaataatacttcctagtagttactataatatacatactctctaGTTTTCCCAGTCACGACGTT |
| CDC15-TET-F | tttgaagacaacaaaccaaatggaaagatgatcactaaagtagccttagatatatagtttttataaGTAATACGACTCACTATAGG |
| CDC15-TET-R | tggctcgtgtgtaaagttgtcctttcatttaaagcttctgtcttcaaatcaaaatcatttaaaaccatCTAGTTTTCTGAGATAAAGCTG |
| CDC15-TET-V5-R | gctcgtgtgtaaagttgtcctttcatttaaagcttctgtcttcaaatcaaaatcatttaaaaccatCGTAGAATCGAGACCGAGG |
| TET-V5-R | CGTAGAATCGAGACCGAGGAGAGGGTTAGGGATAGGCTTACCCATTTTCTAGTTTTCTGAGATAAAGCTG |
| TET-F | GTAATACGACTCACTATAGGG |
| CDC15-UAU-F | gctttaaatgaaaggacaactttacacacgagccagaatgcaagttcgaagaatggatcgaacaaagaatctgttcttGTTTTCCCAGTCACGACGTT |
| CDC15-UAU-R | caccagtaaaagttctgtagtgacttcttcaatcaacaatgagctttgtctcagctttaatgactgcaatgtatTGTGGAATTGTGAGCGGATA |
| TUB1-GFP-F | tggctgctttagagagagattatattgaagttggtactgattctttccctgaagaagaagaagaatatGGTGGTGGTTCTAAAGGTGAAGAATTATT |
| TUB1-NAT1-R | tagtaaataataaaaatgattatgtagaaaaaaaaaagaatagaaatcagaaaaaattataatgaaaaacccagacctCGTTAGTATCGAATCGACAGC |
| CDC15-GFP-F | cattaaagctgagacaaagctcattattgattgaagaagtcactacagaacttttactggtgctaaagGGTGGTGGTTCTAAAGGTGAAGAATTATT |
| CDC15-NAT1-R | tcgttgcttctctttgtttttttgctactattataaaataatacttcctagtagttactataatatacatactctCGTTAGTATCGAATCGACAGC |
| TUB4-RFP-F | gatgacctagaagatggtggtggtaatggtaatggttataacaatatagatgatgcagatatgggtataGGTGGTGGTGATAACACTGAAGATGTTATT |
| TUB4-URA3-R | tgactccacaaccacaaaagttattcctcaactcggacattatctttttattctatatacatttaaccttctcttacaTCTAGAAGGACCACCTTTGATTG |
| CDC3-GFP-F | acaaaaattattaccacaagacccaccagcacaaccagctccacaaaagagtcgtaaaggatttttacgtGGTGGTGGTTGTAAAGGTGAAGAATTATT |
| CDC3-NAT1-R | tactgacaatttttatacatcacaatatcaaattaaacaaacagattaacaaacaaataaactaaattaagttacataCGTTAGTATCGAATCGACAGC |

**^a^** gene specific sequences are in lower case
